# Torsion bottle, a very simple, reliable, and cheap tool for a basic scoliosis screening

**DOI:** 10.1186/s13013-018-0150-6

**Published:** 2018-02-06

**Authors:** Michele Romano, Matteo Mastrantonio

**Affiliations:** ISICO (Italian Scientific Spine Institute), Via Bellarmino 13/1, Milan, Italy

**Keywords:** Prominence, Scoliometer®, Measurement

## Abstract

**Background:**

One of the reasons that make scoliosis a disease that scares so much the parents is its specific characteristic of being difficult to detect on its onset.

The aim of this paper is to check the possible usefulness of a simple tool (the torsion bottle) that has been developed with the aim to offer an instrument for home use by parents but also for screening purposes in the low-income countries.

**Methods:**

Study design: retrospective analysis to evaluate intra-operator reliability of the tools and inter-operator repeatability using the torsion bottle.

For the first and the second part of the study, 35 subjects were measured.

The goal of the first experiment was to evaluate the reliability of the torsion bottle to identify all individuals who experienced a thoracic or lumbar prominence equal or greater than 7°.

The secondary aim was to verify the reliability of blinded inter-operator assessments, performed with the torsion bottle by two physiotherapists on the same patients.

**Results:**

The reliability of the assessments of the torsion bottle has been performed with the Kappa statistic to evaluate the measurement agreement.

The results have shown that the intra-operator reliability of the tool is very high between the measurements collected with the scoliometer® and those collected with the torsion bottle (kappa = 0.9278; standard error = 0.7094).

The data of the second part of the study show that the inter-operator reliability is good (kappa = 0.7988; standard error 0.1368).

**Conclusion:**

The collected data showed that the torsion bottle revealed itself as an efficient tool to execute a basic screening to identify the presence of a prominence in a significant group of adolescents.

## Background

One of the reasons that make scoliosis a disease that scares so much the parents is its specific characteristic of being difficult to detect on its onset.

Too often, it is possible to recognize the appearance of the spinal curves when the disease is already severe.

The reason is due to the typical, slow, and insidious debut of the symptoms.

Being able to identify early the first signs of misalignment of the spine is one of the most effective treatment keys.

Obviously, a frequent monitoring of the clinical signs of scoliosis is the simplest solution [1–3].

One of the most important clinical signs of this disease is the prominence [4]. The presence of the prominence is internationally recognized as an indisputable element of the pathology [5–7].

For the prominence assessment, a scoliometer® is usually used [8, 9].

The scoliometer® is a professional tool used in the medical field by physicians and physical therapists.

Obviously, in a domestic field, the scoliometer® is not available.

For this reason, a very simple tool called torsion bottle, cheap and easy to create, has been developed.

The aim of this paper is to check the possible usefulness of this simple tool (the torsion bottle) that has been developed with the aim to offer an instrument for home use by parents but also for screening purposes in the low-income countries.

## Methods

Study design: retrospective analysis to evaluate intra-operator reliability of the tool and inter-operator repeatability.

The study was divided into two parts. The main aim was to compare the measurements collected with the torsion bottle and the same measurements collected with the scoliometer® (gold standard) in a population of patients accessing our clinic for the rehabilitation treatment, measured according to our standard evaluation protocol.

The secondary aim was to verify the reliability of blinded inter-operator assessments, performed with the torsion bottle by two physiotherapists on the same patients. These double-blinded measurements were taken periodically for a short time interval in our clinic to verify measurement accuracy in the everyday clinical activities.

### The tool

The torsion bottle is composed of a plastic water bottle of 500 ml.

The perfect accuracy of the amount of water to be used is not an essential element. On the outer surface of the bottle, it will be marked with a series of four landmarks that will be essential for its use. These landmarks will be marked only after the bottle is partially emptied and carefully closed.

These landmarks will be adjusted only to the particular amount of water contained in that bottle. In any case, it is recommended to fill approximately half of the bottle.

The torsion bottle is used in a horizontal position as a scoliometer®.

The rationale of the preparation of the torsion bottle is the variation of the position of the little quantity of water inside the bottle, when the bottle is tilted.

**Fig. 1:**
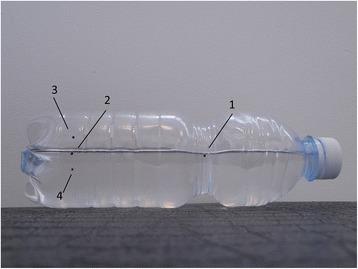
The four landmarks marked on the torsion bottle

**Fig. 2:**
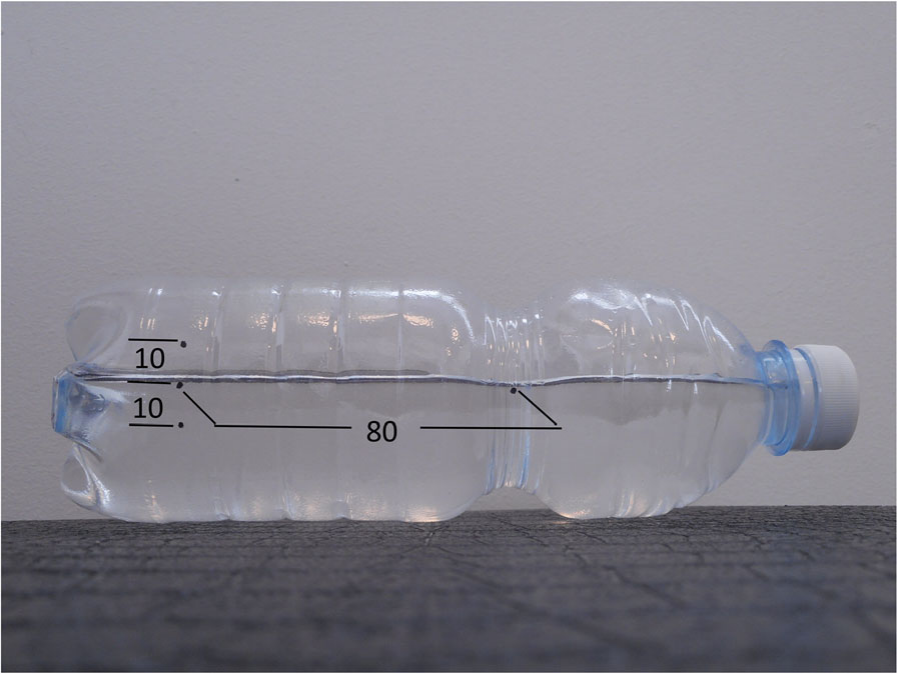
The distances (in mm) between the landmarks

The essential reference signs are:

- Landmarks 1 and 2 which should coincide with the water surface.
- Landmark 3 is the limit of the 7° of the bottle’s tilt, and it represents the cutoff to identify the subjects with a prominence less, equal, or greater than this value (Fig. 1).
- Landmark 2 is marked at a distance of 80 mm with respect to landmark 1.
- Landmark 3 is marked 10 mm from the landmark 2 in the orthogonal direction (Fig. 2).

### How to use the torsion bottle

**Fig. 3:**
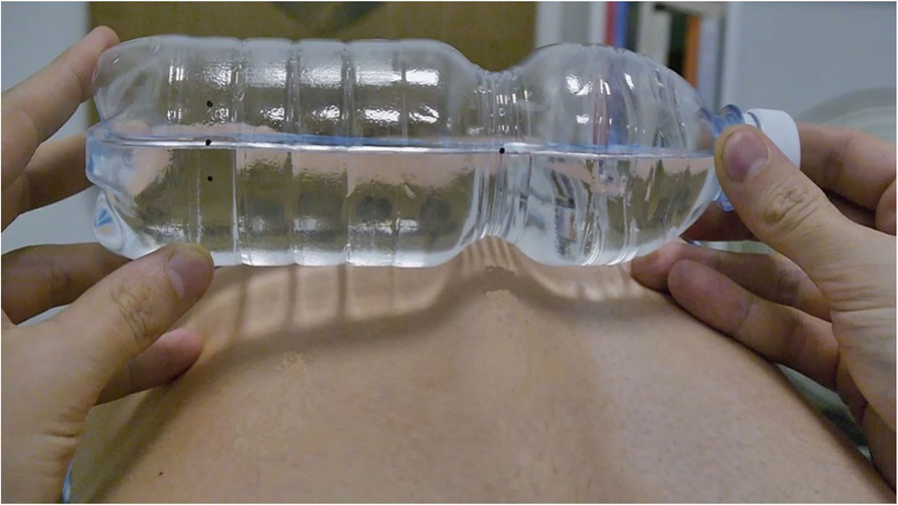
Use of the torsion bottle. Positioning of the tool

**Fig. 4:**
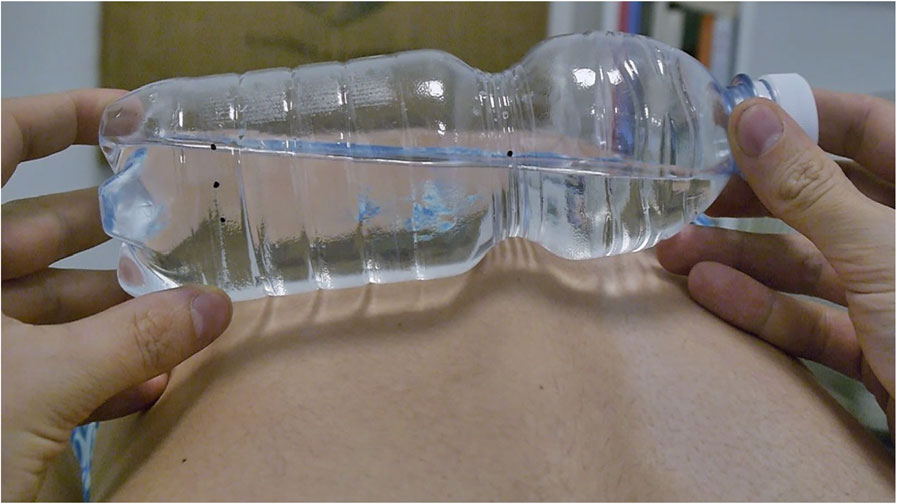
Tilt of the torsion bottle toward the concavity of the curve

**Fig. 5:**
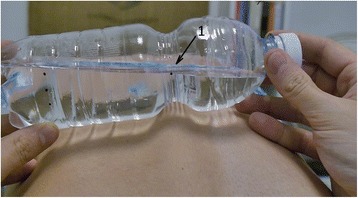
Rotation of the torsion bottle and realignment of the water surface with the landmark number 1

**Fig. 6:**
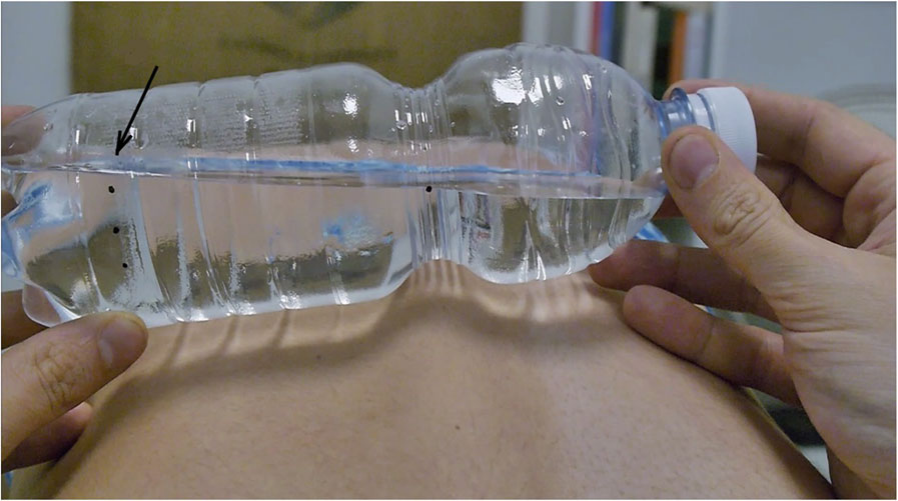
Check of the position of the water surface respect the landmark number 3

How to use the torsion bottle is as follows:

- Forward bending of the patient’s trunk to perform an Adam test [10].
- The torsion bottle positioned crosswise on the patient’s back with the landmark 1 positioned over the line of bony prominences and the water surface coinciding with landmarks 1 and 2 (Fig. 3).
- Tilt the bottle until it touches the patient’s back (Fig. 4).
- Rotate the bottle until the surface of the water coincides with the position of landmark 1 (Fig. 5).
- Observe the position of the surface of the water with respect to landmarks 3 or 4 (Fig. 6).

### Instructions for the preparation and the use of the torsion bottle

https://drive.google.com/file/d/0B2u_ASmUxo0_bjBiQ0ptTUl5M3M/view?ts=57d16f0e.

The water surface slope, with respect to the reference point, corresponds precisely to 7125°.

The following table shows the variability linked to the potential errors in the mark of the reference points (Table 1).

**Table 1:** Distances between reference points and relative degrees

Dots 1-2 (cm)	Dots 2-3 (cm)	Degree
8	1	7,125
8	0,7	5,001
8	0,8	5,711
8	0,9	6,419
7,9	0,7	5,064
7,9	0,8	5,782
7,9	0,9	6,499
7,8	0,7	5,128
7,8	0,8	5,856
7,8	0,9	6,582
7,7	0,7	5,194
7,7	0,8	5,932
7,7	0,9	6,667
8,1	0,7	4,939
8,1	0,8	5,641
8,1	0,9	6,340
8,2	0,7	4,879
8,2	0,8	5,572
8,2	0,9	6,263
8,3	0,7	4,821
8,3	0,8	5,505
8,3	0,9	6,189
8	1,2	8,531

For the first part of the study, 35 subjects were measured. This group included consecutive adolescents of both genders who attend a specialized clinic for the conservative treatment of scoliosis to perform their medical examinations.

The goal of the experiment was to evaluate the reliability of the torsion bottle to identify all individuals who experienced a prominence equal or greater than 7°.

The presence of a prominence higher than 7° is considered a clinical sign very likely linked to a scoliosis that requires a medical treatment [1].

The assessment was performed by the same evaluator.

The evaluator asked the patient to bend the trunk forwards to perform an Adam test.

As a first action, he performed the measurement with the torsion bottle, and in a second time, the measurement with the scoliometer® precisely performed on the same point of the trunk.

For the second part of the study, the assessors measured the prominences of 35 consecutive patients who attended the same clinic for a session of conservative treatment based on Physioterapic Scoliosis Specific Exercises (PSSE).

Measurements were collected blinded by two physiotherapists and processed by a third operator.

The first assessor performed the first evaluation.

The evaluator asked the patient to bend the trunk forwards to perform an Adam test.

The assessor used the torsion bottle to measure the prominence.

**Fig. 7:**
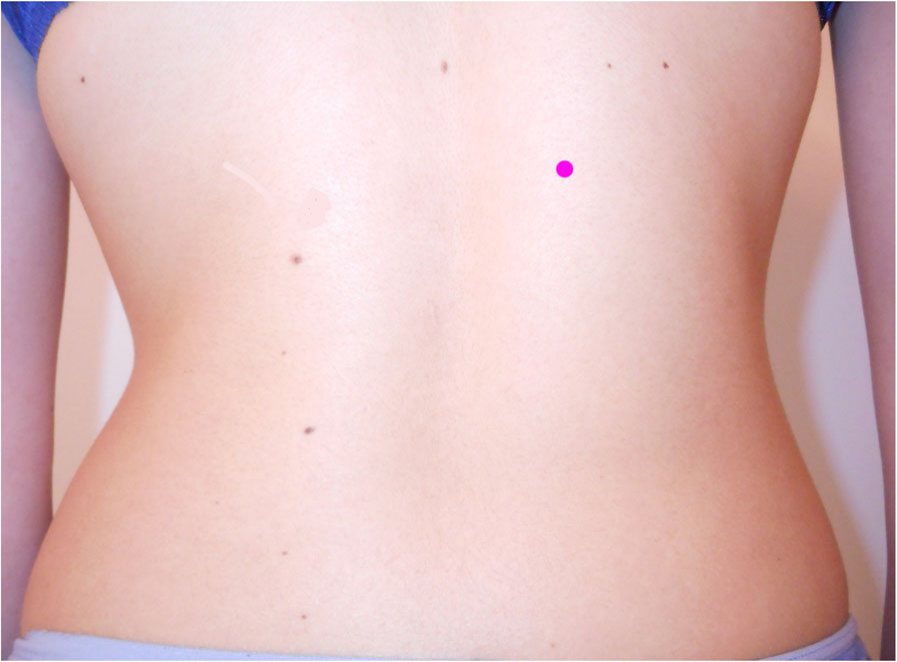
Landmark on the prominence to made the measure on the same point

The evaluator made a small sign at the back where the measurement had been taken, to enable the second prominence assessment on the same spot (Fig. 7).

The subject was accompanied to another room to carry out a second evaluation performed by the second evaluator.

The data collected in two different files have been handed over to a third operator who performed the comparison and the statistical tests.

## Results

The following table shows the detail of the measurements taken for the first part of the study (Table 2):

- Column 1: gender of the subject
- Column 2: the result of the measurement performed with the torsion bottle and the positive or negative hypothesis of the presence of a prominence equal to or greater than 7°
- Column 3: the measurement of the angle trunk rotation performed with the scoliometer®
- Column 4: the agreement of the measurement performed with the torsion bottle and the scoliometer®

**Table 2:** Concordance between Torsion Bottle and Scoliometer assessments

Gender	Torsion Bottle Assessment	Scoliometer Assessment	Concordance
Female	Positive	13	YES
Female	Positive	8	YES
Female	Positive	11	YES
Female	Positive	7	YES
Female	Negative	5	YES
Female	Positive	10	YES
Female	Positive	10	YES
Female	Positive	7	YES
Male	Negative	3	YES
Female	Positive	8	YES
Female	Positive	8	YES
Female	Positive	7	YES
Female	Positive	11	YES
Female	Positive	8	YES
Female	Negative	3	YES
Female	Negative	8	NO
Female	Positive	7	YES
Female	Positive	9	YES
Male	Positive	12	YES
Male	Positive	9	YES
Female	Positive	8	YES
Female	Positive	8	YES
Female	Positive	8	YES
Female	Negative	4	YES
Female	Positive	7	YES
Female	Positive	10	YES
Female	Negative	5	YES
Female	Positive	10	YES
Female	Negative	5	YES
Male	Negative	6	YES
Female	Negative	6	YES
Female	Positive	8	YES
Female	Positive	7	YES
Female	Negative	5	YES
Female	Positive	11	YES

Table 3 shows the details of the measurements of the second part of the study:

- Column 1: gender of the subject
- Column 2: results of the first assessor
- Column 3: results of the second assessor

**Table 3:** Agreement between Torsion Bottle assessment performed by blinded assessors

Gender	Assessor 1	Assessorr 2
Female	Positive	Positive
Female	Positive	Positive
Female	Positive	Positive
Female	Positive	Positive
Female	Positive	Positive
Female	Negative	Negative
Female	Positive	Positive
Male	Positive	Positive
Female	Positive	Positive
Female	Positive	Positive
Female	Positive	Positive
Male	Negative	Negative
Male	Positive	Positive
Male	Positive	Negative
Female	Positive	Positive
Female	Positive	Positive
Female	Positive	Positive
Female	Positive	Positive
Female	Positive	Positive
Female	Positive	Positive
Female	Negative	Negative
Female	Positive	Positive
Female	Positive	Positive
Female	Positive	Positive
Female	Positive	Positive
Female	Positive	Positive
Female	Positive	Positive
Male	Negative	Negative
Female	Positive	Positive
Female	Positive	Positive
Female	Positive	Positive
Female	Negative	Positive
Female	Positive	Positive
Female	Negative	Negative
Female	Positive	Positive

The reliability of the assessments of the torsion bottle has been performed with the Kappa statistic to evaluate the measurement agreement.

The results have shown that the intra-operator reliability of the tool is very high between the measurements collected with the scoliometer® and those collected with the torsion bottle (kappa = 0.9278; standard error = 0.7094).

The data of the second part of the study show that the inter-operator reliability is good (kappa = 0.7988; standard error 0.1368).

## Discussion

The collected data showed that the torsion bottle revealed itself as an efficient tool to execute a basic screening to identify the presence of a prominence in a significant group of adolescents.

The basic scoliosis screening is a very simple process but is not performed regularly because, in many countries, the health policy of this disease is based on the concept of “wait and see.” Unfortunately, this does not help in prevention but too often leads to surgical intervention, when the severity of the disease is serious.

This torsion bottle has not been conceived for a professional use because the torsion bottle does not quantify the degree of the prominence, unlike the scoliometer®.

It is considered more useful for a domestic use after having trained the parents. This makes it possible to achieve greater awareness and more frequent monitoring in situations of young patients at risk of evolution.

## Conclusion

The torsion bottle is useful to perform a pre-investigation of the presence of a prominence, permitting a simplified assessment of its value thus directing to a specialist for a medical assessment.

Another use of the torsion bottle may be provided for screening to be carried out in countries where due to the low-income problems it is difficult to find a scoliometer® or where the presence of health professionals is not so widely distributed.

The water plastic bottles do not have the same shape in all countries. In some countries, it is typical to find this small bottle with a hollow in the upper part (Fig. 1).

This hollow is useful because it avoids a contact with the bony prominences of the spinous processes. When such a tool is not available and it is necessary to use a bottle with a straight profile, it is important to pay attention to the spinous processes to avoid that the contact with the bottle falses the measurement.
